# Recombinant HBsAg of the Wild-Type and the G145R Escape Mutant, included in the New Multivalent Vaccine against Hepatitis B Virus, Dramatically Differ in their Effects on Leukocytes from Healthy Donors In Vitro

**DOI:** 10.3390/vaccines10020235

**Published:** 2022-02-03

**Authors:** Maria V. Konopleva, Vera N. Borisova, Maria V. Sokolova, Tatyana A. Semenenko, Anatoly P. Suslov

**Affiliations:** 1 Federal State Budget Institution “National Research Center for Epidemiology and Microbiology Named after Honorary Academician N.F. Gamaleya” of the Ministry of Health of the Russian Federation, 123098 Moscow, Russia; sokolova_mariya_gamaleya@mail.ru (M.V.S.); semenenko@gamaleya.org (T.A.S.); Suslov.Anatoly@gmail.com (A.P.S.); 2 CJSK “RPC COMBIOTECH”, 117997 Moscow, Russia; borisova@combiotech.com

**Keywords:** HBV, HBsAg, escape mutant, G145R mutant, hepatitis B vaccine, Bubo^®^-Unigep, PBMC, antibodies, cytokines, surface markers, immune response

## Abstract

Immune-escape hepatitis B virus (HBV) mutants play an important role in HBV spread. Recently, the multivalent vaccine Bubo^®^-Unigep has been developed to protect against both wild-type HBV and the most significant G145R mutant. Here, we compared the effects of recombinant HBsAg antigens, wild-type and mutated at G145R, both included in the new vaccine, on activation of a human high-density culture of peripheral blood mononuclear cells (PBMC) in vitro. The antigens were used either alone or in combination with phytohemagglutinin (PHA). None of the antigens alone affected the expression of CD40, HLA-DR or CD279. Wild-type HBsAg enhanced CD86 and CD69 expression, and induced TNF-α, IL-10, and IFN-γ, regardless of the anti-HBsAg status of donor. In the presence of PHA, wild-type HBsAg had no effect on either of the tested surface markers, but increased IFN-γ and IL-10 and inhibited IL-2. In contrast, the G145R mutant alone did not affect CD86 expression, it induced less CD69, and stimulated IL-2 along with lowering levels of TNF-α, IL-10, and IFN-γ. The G145R mutant also suppressed PHA-induced activation of CD69. The dramatic differences in the immune responses elicited by wild-type HBsAg and the G145R mutant HBsAg suggest distinct adaptive capabilities of the G145R mutant HBV.

## 1. Introduction

Few diseases of a viral nature have the same global significance as hepatitis B virus (HBV). Approximately one-third of the world population is infected with HBV, including about 360 million people infected chronically [1]. Historically, the World Health Organization (WHO) Western Pacific Region (WPR) has had the world’s highest prevalence of chronic HBV infection (greater than 5%) [2]. Due to the clinical complications of the infection, such as cirrhosis of the liver and hepatocellular carcinoma, about 1 million deaths occur each year [1]. Globally, HBV-related liver disease represents the seventh highest cause of mortality worldwide [2].

In 2016, the WHO committed to eliminating viral hepatitis as a public health threat by 2030. However, considering the level of efforts currently deployed to tackle the disease, these targets are highly unlikely to be achieved over the next decade, especially in resource-limited settings. By 2017, approximately 70% of countries/regions worldwide had formulated a national plan to achieve the WHO goal of elimination of viral hepatitis, but fewer than 50% of the countries/regions had secured funding to support those plans, and even in those that did, the amount of funding did not cover the entire plan. Rates of hepatitis B diagnosis are very low, averaging 8% globally. The proportion of patients with hepatitis B who are receiving treatment is 5%. The currently available antivirals—nucleos(t)ide analogues (NUCs)—cannot eradicate the virus, and only induce viral suppression.

By the end of 2018, a hepatitis B infant vaccine was introduced in 189 countries/regions, and the global coverage with three doses of the vaccine was estimated at 84%. However, the estimates remain insufficient (76%) in Africa, and HBV birth-dose vaccine coverage globally is still very low (38%), especially in Africa, where only 11% of newborn babies receive the HBV birth dose within 24 h after birth, as recommended. The Global Alliance for Vaccine and Immunization (GAVI) has still not confirmed its support for HBV birth-dose provision in Low-to-Middle-Income Countries (LMICs), although the success of vaccination is obvious. For example, in 1990, more than 8% of children of at least 5 years of age in the WPR had chronic HBV infection, and thanks to the vaccination program, 5-year-old children had a prevalence of 0.93% in 2017. Regarding the elimination of HBV, Taiwan was the first country to implement universal hepatitis B vaccination in 1986, and the overall HBV carriage rate in the vaccinated cohort was reduced to 0.5% in 2016 [2].

At present, the most widely used hepatitis B vaccines are second-generation vaccines comprising S-HBsAg of ayw, adw and adr subtypes expressed in different yeast strains, and formulated with either aluminum hydroxide or aluminum hydroxyphosphate sulfate as adjuvants. Second-generation vaccines are mostly HBsAg-monovalent (including one of the aforementioned wild-type subtypes), although some are HBsAg-bivalent (including a combination of S-HBsAg ayw and adw subtypes or S-HBsAg and M-HBsAg of adr subtype) [3,4]. Furthermore, second-generation HBV vaccines are also used as components of combined vaccines containing antigens of other pathogens, including hepatitis A virus, *Bordetella pertussis*, *Corynebacterium diphtheriae*, *Clostridium tetani*, *Poliovirus hominis*, and *Haemophilus influenzae* type B. Additionally, there is a hepatitis B vaccine containing S-HBsAg synthesized in *Hansenula polymorpha*, formulated with a Toll-like receptor 9 (TLR 9) agonist, cytidine–phosphate–guanosine oligodeoxynucleotide (CpG-ODN) 1018, as an adjuvant. Furthermore, third-generation vaccines are available, which utilize different yeast expression systems and mammalian cell lines for the vaccine production. These vaccines comprised M-HBsAg and L-HBsAg proteins in the presence or absence of S-HBsAg. Third-generation vaccines can be HBsAg-monovalent or bivalent and include HBsAg of adw and ayw subtypes. Moreover, chimeric HBV vaccines are under development. In chimeric vaccines, envelope proteins of HBV are exploited as carrier platforms for foreign antigenic sequences by introducing N- or C-terminal extensions, N-terminal extensions in addition to substitutions of the S-HBsAg N-terminal sequence, by replacing the M-HBsAg preS2-domain, by insertions into the external loop region including replacing antigenic determinants, or by replacing S-HBsAg-specific cytotoxic T-lymphocyte (CTL) epitopes. So far, only one such vaccine has been licensed, which includes the malaria antigen. Therapeutic HBV vaccines have also been developed during the past two decades. Most of those include HBcAg and the preS1 region of HbsAg (or its individual epitopes). The S-HbsAg region is not always included in the composition of the candidate vaccine antigen. An active search for new adjuvants for HBV therapeutic vaccine candidates is ongoing [3]. The development of polyvalent vaccine formulations that include an HBV vaccine for infants and children is an important step toward improved vaccine coverage worldwide [2].

The high prevalence of hepatitis B virus (HBV) infection is largely due to HBV survival under conditions of immunological pressure, which is facilitated by a variety of mechanisms which evolved along with the virus’s evolution [5,6]. HBV affects both the innate and adaptive arms of the immune system and employs two essential strategies: the “invisible virus” (or the “stealth virus”) and immunosuppression [7,8,9]. The following mechanisms contribute to HBV’s “stealth” strategy: distinct features of HBV replication that prevent virus recognition by the innate immune system [6,10], the emergence of immune-escape and vaccine-escape mutants [7,9,11], the virus hiding in host cells and tissues, ensuring its inaccessibility for T cells [6,7,12,13], and overproduction of subviral particles that act as traps for antivirus antibodies [14]. On the other hand, the immunosuppression strategy implemented by HBV appears to be predominantly based on viral apoptotic mimicry [8,14,15]. The latter results in dysfunction of natural killer (NK) cells and natural killer T (NKT) cells [5], inactivation of dendritic cell functions, and inhibition of the adaptive immune system [5,9,16]. At the same time, the interaction between HBV and the entire immune system may be in the state of “dynamic balance” that is under the influence of various factors [5,8].

Whereas the aforementioned mechanisms have been proposed for wild-type HBV, the immunological properties of escape mutants have not been explored sufficiently, and most studies have focused on the interaction of mutants with anti-HBsAg antibodies, typically by ELISA [17,18,19,20], and less often using cell lines [21]. However, since pathogenetic changes caused by HBV mutants can differ from those of wild-type HBV, investigation into the effects of HBV mutants on various arms of the immune system is essential for both fundamental and applied science [6].

HBV mutants with clinical implications have been found worldwide, indicating their potential to spread and develop their own epidemiological properties. A large-scale assessment of the geographic prevalence of immune-escape HBV mutants showed that overall they were detected in 10.7% of the sequences, predominantly in North America (16.3%), East Asia (12.3%), and Southeast Asia (12.0%), and with lower frequency in Central Africa (0.0%), East Africa (1.4%), and the Caribbean (1.6%). HBV genotype B showed the highest mutation rate (14.7%), followed by genotypes C (11.2%), G (10.0%), D (9.3%), A (7.5%), E (5.1%), and F (2.0%), while genotype H did not show such mutations. Across different genotypes, the most frequent substitutions were I/T126S (1.8%), G145R (1.2%), M133T (1.2%), Q129R (1.0%), I/T126A (0.8%), and P120T (0.8%). These major mutations were more common in genotypes A (P120T, 3.2%), B (I/T126A, 2.8%; Q129R, 2.2%), C (I/T 126S, 3.9%; M133T, 1.9%), and G (G145R, 5.0%) [22]. Studies in Taiwan showed that escape mutations were detected at a frequency of 28.1% among vaccinated children, with the proportion of the G145R mutant being 15.4% [23]. In Japan in 2016, in a cohort of children born to HBV-carrier mothers who became HBV carriers despite immunoprophylaxis, the frequency of the G145R mutant ranged from 0.54% to 6.58%. In this control cohort, the frequency of the G145R mutant ranged from 0.42% to 4.1% [24]. These findings stress the ongoing spread of the G145R escape mutant despite large-scale vaccination, especially among children. It is noteworthy that in the work of Araujo et al., the G145R mutant was found only in genotypes G (5.0%), D (2.5%), C (2.1%), and B (0.2%) [22], while Datta et al. reported the G145R mutant of the HBV Ae/A2 subgenotype, and in most subjects this mutant was detected only in leukocytes [12]. The true prevalence of escape mutants can be underestimated, due to insufficient sensitivity of detection as well as accumulation of mutants in special-risk groups of people. For example, deep sequencing of HBV by the next-generation sequencing (NGS) method in hematological cancer patients revealed an exceptionally high frequency of mutations in patients with leukemia (up to 93.4%) and lymphoma (up to 85.0%), and the mutations were noticeably heterogeneous. Moreover, in 6.3% of leukemia and 5.0% of lymphoma cases, more than 15 mutations in S-HBsAg were detected in the same sample, and most of those were escape mutations [25].

The effectiveness of HBV vaccines against HBV mutants has been questioned repeatedly. For example, immunization of chimpanzees with licensed recombinant HBV vaccines stimulated production of broadly reactive anti-HBsAg antibodies that protected against infection with the G145R mutant [26]. However, it was later shown that the single-substitution mutant G145R is unstable in chimpanzees, as it reverts to the wild type during viremia. In contrast, the HBV variant with three mutations in the polymerase gene (rtV173L + rtL180M + rtM204V), also generating the G145R mutation in S-HBsAg due to overlapping reading frames, was stable. Evaluation of the vaccine’s effectiveness against hepatitis B in chimpanzees showed the absence of sterilizing immunity against such a polymerase mutant [27]. In addition, studies of sera from vaccinated people have shown that the widely used vaccines provide virtually no cross-reactive protection against the G145R mutant in the virus neutralization assay, in contrast to, for example, another immune-escape mutant S143L [28]. Taken together, the data, along with results of mathematical models [29,30], indicate that the G145R mutant may be epidemiologically dangerous. Although several patents for candidate vaccines protecting against the G145R mutant have been filed [31,32,33], none of those have been further developed and approved.

The main reason for the ineffectiveness of postvaccination antibodies against the G145R mutant is the mutant’s distinct antigenic properties. As shown by Waters et al. [17], monoclonal antibodies that recognize peptides containing 139–147 amino acid (aa) and 124–137 aa residues of HBsAg of subtypes ay and ad, either did not bind to the G145R mutant at all or were required at 10 times higher concentrations compared with wild-type HBsAg [17]. In contrast, monoclonal antibodies recognizing the mutant G145R HBsAg did not bind to HBsAg containing other substitutions [34]. When assessing the interaction of an anti-HBsAg monoclonal antibody with wild-type HBV and the G145R mutant by the Ouchterloni double immunodiffusion method, a “cross” where the precipitation lines intersect was observed, indicating that the antigenic determinants are not identical [35]. Furthermore, endocytosis of full-length HBsAg-specific IgG inhibited the secretion of wild-type but not the G145R mutant HBsAg and HBV virions from cell lines [21]. Computer simulations proposed that the G145R substitution results in an insertion of a new β-fold in the HBsAg α-determinant and alters the orientation of the transmembrane segments of the protein, which in turn changes the membrane topology of HBsAg. Moreover, these modifications lead to a loss of a part of the α-helical structure of HBsAg [36]. Hence, the G145R mutation can increase the rigidity, compactness, and aggregation potential of the α-determinants, affecting the immunogenicity and secretion of HBsAg [36,37] as well as the morphogenesis of virions [38]. For example, the cell line transfected with the G145R mutant expressed significantly less L-HBsAg, whereas the expression of S-HBsAg did not change. Additionally, the expressed virus particles were less stable when interacting with the NP-40 detergent [37]. Electron microscopy revealed unusual oval-shaped 60–70 nm HBV structures in serum and 100–200 nm structures in a preparation of the purified G145R mutant, which were not seen in samples of wild-type HBV or an S143L escape mutant [38].

There is currently no registered vaccine globally that is active against the G145R escape mutant. The urgency of the problem, however, dictates the need for developing a vaccine that would elicit a protective humoral immune response against both individual HBV subtypes (ay and ad) and epitopes of the G145R and other escape mutants. It is expected that by expanding the specificity of the immune response, such a new-generation, multivalent vaccine will significantly increase the effectiveness of vaccination against HBV [4]. Given the aforementioned features of the G145R mutant HBsAg folding, the recombinant analogue of the antigen included in the vaccine should have the same biological activity.

Previously, we have selected a recombinant analogue of the G145R mutant that is almost identical to the natural protein [39]. This was achieved using a serological fingerprinting method using a panel of 10 monoclonal antibody conjugates. A set of recognition defects characteristic of the natural G145R mutant detected in sera of chronic HBsAg carriers was determined, and the depth of these defects was assessed semiquantitatively using a four-step scoring system. Further assessment of recombinant antigens with the G145R mutation using the same technique demonstrated that not all of them reproduced the natural serological profile of the given mutant. This was primarily due to the conditions of antigen expression and purification [39]. For example, treatment of recombinant HBsAg (rHBsAg) with urea should be avoided [17], since this compound, as well as other chaotropic salts, can disrupt the structure of the HBsAg protein [40].

The rHBsAg (genotype D, ayw) that was selected based on the antigenic similarity [39] was expressed in the yeast *H. polymorpha* and formed virus-like particles, as confirmed by electron microscopy and gel filtration chromatography [41]. This antigen was immunogenic in mice and sheep. The analysis of the spectrum of postvaccination antibodies was carried out both against the immunogen and the natural HBV G145R mutant. In the latter case, researchers used sera of chronic HBsAg carriers that were characterized by deep sequencing and shown to contain the G145R mutation in HBV adw3 and ayw2 genotype D subtypes (ENA ERZ377006 and ENA ERZ377011) with 99% homogeneity, and applied a method developed for assessing an antibody level specific to different native variants of HBsAg [42]. The results were generally in agreement with the findings of Waters et al. [17]. The recombinant G145R mutant and wild-type HBV differ significantly in immunogenicity and determinant specificity. Thus, HBsAg with the G145R mutation is less immunogenic, requiring large doses and time for the development of an immune response. The rHBsAg with the G145R mutation is capable of eliciting antibodies at the level comparable to the wild-type antigen, and the antibodies that are generated recognize not only the HBsAg G145R mutant but also wild-type HBsAg [39]. Nevertheless, the data suggested that the mechanism of the immune response against the G145R mutant is slightly different than for wild-type HBsAg.

The preliminary selection of rHBsAg containing the G145R mutation, similar to the native analogue in antigenic and immunogenic properties, allowed for developing a component of the hepatitis B vaccine with the G145R escape mutation in HBsAg [39]. In 2019, CJSK “RPC COMBIOTECH” designed a new trivalent vaccine Bubo^®^-Unigep, containing antigens that confer protection against wild forms of HBV subtypes ay and ad, as well as a determinant of serotype ay with the G145R mutation at 10 μg/mL of suspension [4]. Currently, Phase III clinical evaluation of this vaccine is approaching completion.

The aim of the current work was to conduct in-depth in vitro studies of the immunological mechanisms implemented by the G145R mutant, using the recombinant analogue of the natural HBsAg G145R mutant and its wild-type prototype, both included in the Bubo^®^-Unigep vaccine.

## 2. Materials and Methods

### 2.1. Cells and Sera

The study included 20 healthy donors (55% were men and 45% were women). The patients’ buffy coats were obtained from the Federal State Budgetary Institution “National Medical Research Center of Hematology” of the Ministry of Health of the Russian Federation and the State Budgetary Healthcare Institution “Research Institute of Emergency Medicine named after N.V. Sklifosovsky”. Informed voluntary consent was obtained from all patients that participated in the study in accordance with the ethical principles laid down in the World Medical Association Declaration of Helsinki.

Data on vaccination of donors against hepatitis B were not available. Therefore, donors were checked for the presence of a protective titer of antibodies to HBsAg. Sera from 16 donors were tested for antibodies to HBsAg using the VectoHBsAg-antibodies test system (cat. No. D-0562, AO “Vector-Best”, Novosibirsk, Russia), and the donors were divided into two groups according to the level of antibodies to HBsAg: group 1 (*n* = 7), <10 mIU/mL and group 2 (*n* = 9), >10 mIU/mL.

### 2.2. rHBsAg

The studies used wild-type rHBsAg of the ayw2 subtype and rHBsAg with the G145R mutation of the ayw2 subtype, both expressed in the yeast *H. polymorpha* (CJSK “RPC “COMBIOTECH”, Moscow, Russia) [4,39,41,42]. The purity of the antigen preparations, determined by sodium dodecyl sulphate–polyacrylamide gel electrophoresis followed by Coomassie staining, was more than 85%.

### 2.3. Immunophenotyping of PBMC

The immunophenotyping of the donor PBMC was performed by the direct immunofluorescence method using the “7-Color Immunophenotyping Kit” (Miltenyi Biotec, Bergisch Gladbach, Germany). Sample preparation was carried out according to the manufacturer’s instructions. Cells stained with immunofluorescence-labelled antibodies were analyzed using a MACSQuant Analyzer 10 flow cytometer (Miltenyi Biotec, Bergisch Gladbach, Germany), to determine the following cell subpopulations: CD45^+^ (leukocytes), CD45^+^ CD3^+^ (T cells), CD45^+^ CD3^+^ CD4^+^ (T-helper cells), CD45^+^ CD3^+^ CD8^+^ (CTL), CD45^+^ CD19^+^ (B cells), CD45^+^ CD14^+^ (monocytes), SSC^low^CD45^+^ CD14^-^CD16^+^ CD56^+^ CD3^−^ (NK-cells), SSC^high^CD45^+^ CD14^−^CD16^−^ (eosinophils), SSC^high^CD45^+^ CD14^−^CD16^+^ (neutrophils).

The results of immunophenotyping of PBMC from healthy donors [43] as well as the reference values used in clinical practice were used as reference values in the current study.

### 2.4. Stimulation of PBMC with rHBsAg

PBMC were isolated from the buffy coats using a Ficoll density gradient. The buffy coat was first diluted 5 times using Dulbecco’s Phosphate Buffered Saline (DPBS) without calcium and magnesium (Capricorn Scientific, Ebsdorfergrund, Germany) and then layered over Ficoll-Pague Plus (GE Healthcare, Chicago, IL, USA) in a ratio of 3:4 and centrifuged for 35 min at 400× *g*. The ring of mononuclear cells was collected and the cells were washed three times with DPBS at 200× *g* for 10 min. The isolated PBMC were resuspended in the complete culture medium containing RPMI-1640 with HEPES (cat. No. C350p, PanEco, Moscow, Russia), 2 mM L-glutamine (cat. No. F032, PanEco, Moscow, Russia), 1% sodium pyruvate (Capricorn Scientific, Ebsdorfergrund, Germany), 1% essential amino acids (Sigma Aldrich, St. Louis, MO, USA), 100 μg/mL Normocin (Invivogen, San Diego, CA, USA), and 10% inactivated human serum (AB) (National Research Center for Hematology, Moscow, Russia). Thereafter, the cells were plated into wells of a 24-well culture plate at a high seeding density of 7 × 10^6^/mL in 2 mL of culture medium. PBMC were precultivated for 45 h in the complete medium according to the patent [44]. During the preliminary cultivation, the medium was changed every 20 h.

After 45 h of the preliminary cultivation, antigenic stimulation of the PBMC was performed using 20 μg/mL of wild-type rHBsAg (ayw2) or 20 μg/mL of rHBsAg with the escape mutation G145R (ayw2). The complete culture medium was used as a negative control, and phytohemagglutinin (PHA) at a concentration of 1.2 μg/mL (Sigma Aldrich, St. Louis, MO, USA) was used as a positive control. The cells were incubated with the antigens for 24 h at 37 °C, 5% CO_2_. In another series of experiments, the cells were stimulated with the antigens in the presence of PHA. In this case, 1.2 μg/mL of PHA and 20 μg/mL of wild-type rHBsAg (ayw2) or 1.2 μg/mL of PHA and 20 μg/mL of rHBsAg with the G145R escape mutation (ayw2) were added simultaneously to PBMC.

The expression of activation markers on the stimulated PBMC was assessed by flow cytometry. In addition, the culture supernatants were collected from the wells and analyzed for the secretion of cytokines IL-2, IL-10, IFN-α, IFN-γ, and TNF-α using test systems manufactured by AO ”Vector-Best” (Novosibirsk, Russia): “Interleukin-2-EIA-Best“ (cat. No. A-8772), “Interleukin-10-EIA-Best” (cat. No. A-8774), “Alpha interferon-EIA-Best” (cat. No. A-8758), “Gamma interferon-EIA-Best” (cat. No. A-8752), “Alpha-TNF-EIA-Best” (cat. No. A-8756).

### 2.5. Evaluation of the Expression of Activation and Depletion Markers on PBMC after Antigenic Stimulation

The expression of activation markers on PBMC after antigenic stimulation was assessed using a multicolor flow cytometry analysis. The PBMC were first incubated for 10 min at 4 °C in the dark with a reagent to block human Fc receptors (Miltenyi Biotec, Bergisch Gladbach, Germany). Subsequently, the PBMC were stained with monoclonal antibodies for 15 min at 4 °C in the dark, and then washed twice with DPBS with 2% fetal bovine serum (HyClone [Cytiva], Marlborough, MA, USA). The stained and washed cells were resuspended in 300 μL of DPBS with 2% fetal bovine serum and immediately analyzed on a MACSQuant Analyzer 10 flow cytometer (Miltenyi Biotec, Bergisch Gladbach, Germany), collecting a total of 100,000 events. The results were processed using MACSQuantify software, version 2.8.

PBMC were stained with three different panels of monoclonal antibodies (Miltenyi Biotec, Bergisch Gladbach, Germany): for T cells, CD4-VioBlue, CD3-VioGreen, CD279 (PD-1)-FITC, CD69-PE, CD45-PerCP-Vio770, CD8-PE-Vio770, and HLA-ABC-APC-Vio-770; for B cells, CD20-VioBlue, CD3-VioGreen, CD86-FITC, CD69-PE, CD40-PE-Vio770, and CD45-APC-Vio770; and for T/B/NK cells, CD20-VioBlue, CD3-VioGreen, HLA-DR-FITC, CD69-PE, CD16/CD56-PE-Vio770, and CD45-APC-Vio770.

For all the three antibody panels used, the following common elements of the gating strategy were applied (see Supplementary Figures S1–S4.): selection of a measurement time interval with a uniform sample delivery, clipping of adherent cells according to the FSC-A/FSC-H diagram, and isolation of lymphocytes using the CD45 marker. Further gating steps varied slightly depending on the antibody panel used. Assessment of T-cell activation with the main panel of antibodies included isolation of T cells by the CD3 marker based on the histogram of the fluorescence intensity, separation of two T-cell subpopulations (CD4^+^ and CD8^+^), and further determination of expression of the markers CD279 (PD-1) and CD69 in them. In addition, the mean fluorescence intensity (MFI) of the HLA-DR marker was determined on T cells using a third panel of antibodies. For both the second and third antibody panels, T cells and B cells were commonly isolated by the CD3 and CD20 markers, respectively. B cells were analyzed for MFI of the CD69, CD40, and CD86 markers using the second panel, and for HLA-DR MFI using the third panel. NK cells were isolated from the gate of CD3^-^CD20^-^ cells using the CD56 marker using the third panel, after which the expression of the CD69 marker was determined in NK cells using a histogram of the fluorescence intensity.

For each multivariate immunophenotype analysis, the compensation was adjusted before measurements in an automatic mode. Gates for activation markers CD69, CD86, CD40, and HLA-DR were set up relative to the isotype controls for the antibodies and negative control samples. The following reagents were used as isotype controls: mouse IgG1-PE-Vio770, REA Control (S)-FITC, mouse IgG2a-FITC, and mouse IgG1-PE (Miltenyi Biotec, Bergisch Gladbach, Germany).

### 2.6. Statistical Analysis

The results were statistically processed using GraphPad Prism v. 6.01 (GraphPad Software, San Diego, CA, USA) software. The data were first examined for normal distribution by the Shapiro–Wilk test. Since many of these tests did not pass, we applied the nonparametric Wilcoxon matched-pairs signed-rank test to determine the significance of differences. Additionally, the nonparametric Mann–Whitney test was used to compare levels of stimulated cytokine production in two unpaired groups of donors with different basic anti-HBsAg levels. For comparison of categorical data (for example, the number of donors that answered to the antigen), Fisher’s exact test was used. The differences between experimental groups were considered statistically significant at *p* < 0.05. Quantitative indicators reflecting the level of expression of activation markers or cytokine concentrations are presented in the paper as M ± m, where M is the mean and m is the standard error of the mean (SEM). Standard deviation (SD) was applied to graphically indicate the ranges of data variation, unless otherwise specified. The results for each individual donor are presented as separate dots on graphs.

## 3. Results

### 3.1. Immunophenotyping of PBMC from Healthy Donors

Immunophenotyping of PBMC obtained from the buffy coats of healthy donors was carried out to identify and reject donors with immune characteristics that do not fit into the clinical reference values (see Table 1).

Overall, the results of the immunophenotyping showed that the average values for 20 donors completely fit into the reference values, despite the fact that these standards were developed to measure blood parameters and not those for isolated PBMCs. The deviation observed in the neutrophil population was most likely due to the loss of these cells during PBMC isolation. Analysis of individual donors demonstrated slight deviations from the control values in some donors, although these deviations were not significant (data not shown).

### 3.2. Evaluation of Activation Markers on PBMC from Healthy Donors after In Vitro Stimulation with rHBsAg

**Figure 1:**
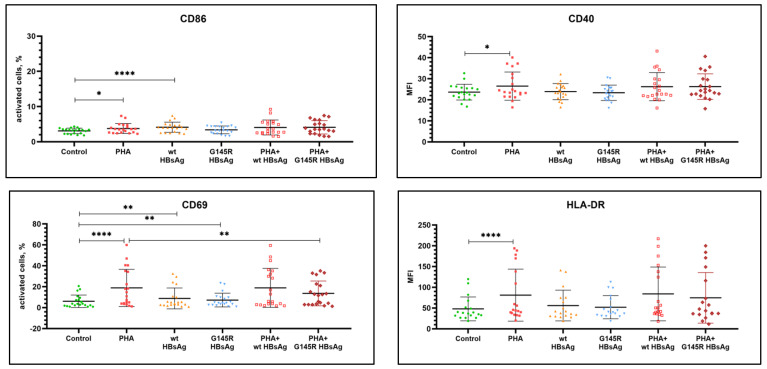
Assessment of surface markers on B cells stimulated with wild-type rHBsAg, the rHBsAg G145R mutant, or PHA, or their combinations. MFI—mean fluorescent intensity. Significant *p* values are reported as asterisks, where * *p* ≤ 0.05, ** *p* ≤ 0.01, and **** *p* ≤ 0.0001.

**Figure 2:**
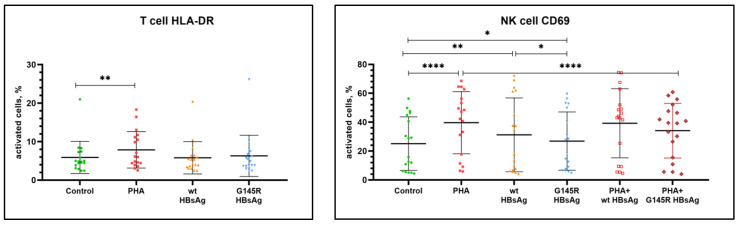
Assessment of surface markers on PBMC stimulated with wild-type rHBsAg, the rHBsAg G145R mutant, or PHA, or their combinations. Significant *p* values are reported as asterisks, where * *p* ≤ 0.05, ** *p* ≤ 0.01, and **** *p* ≤ 0.0001.

**Figure 3:**
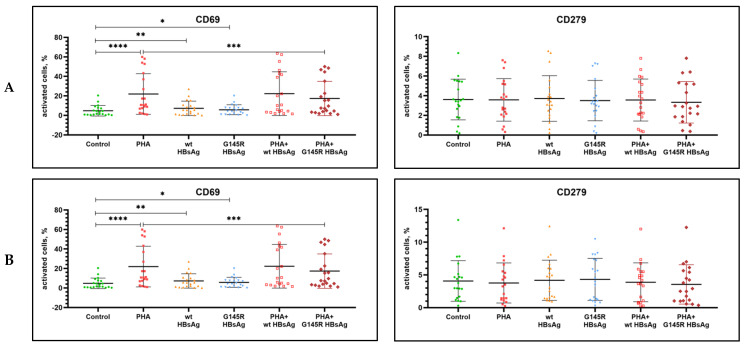
Assessment of surface markers on T cells stimulated with wild-type rHBsAg, the rHBsAg G145R mutant, or PHA, or their combinations. (**A**)—CD4^+^ T cells, (**B**)—CD8^+^ T cells. Significant *p* values are reported as asterisks, where * *p* ≤ 0.05, ** *p* ≤ 0.01, *** *p* ≤ 0.001, and **** *p* ≤ 0.0001.

The activation state of PBMC from healthy donors after antigenic stimulation in vitro was assessed by the level of expression of the following markers: CD69, CD86, CD40, CD279 (PD-1), and HLA-DR. PHA at the concentration of 1.2 μg/mL was used as a positive control, and cells without antigenic stimulation, cultured under the same conditions, were used as a negative control. Statistical analysis of the results was performed to compare to both nonstimulated cells and PHA-stimulated cells. Table 2 and Figure 1, Figure 2 and Figure 3 summarize the data on the expression of activation markers on PBMC after antigenic stimulation with wild-type rHBsAg (ayw2) or rHBsAg with the G145R mutation (ayw2).

The positive control PHA stimulation caused a statistically significant increase in the expression of CD69, CD86, CD40, and HLA-DR on B cells; CD69 on NK and T cells (both CD4^+^ and CD8^+^), and HLA-DR on T cells.

Wild-type rHBsAg caused a statistically significant increase in the expression CD86 and CD69 but no activation of other markers (CD40, HLA-DR) on B cells. Additionally, it caused a statistically significant increase in the expression CD69 on T cells and NK cells. There was no activation of HLA-DR or CD279 on T cells.

rHBsAg with the G145R mutation had no statistically significant effect on the expression of activation markers on PBMC from healthy donors except the expression of CD69 on all tested cells. However, the CD69 activation induced by the rHBsAg G145R mutant was weaker compared with wild-type rHBsAg. The data were significant for NK cells (*p =* 0.0333), and a trend was observed for B cells, CD4^+^ T cells and CD8^+^ T cells (*p =* 0.2774, *p =* 0.5519, and *p =* 0.1075, respectively).

The effects of wild-type rHBsAg (ayw2) and rHBsAg with the G145R mutation (ayw2) on PHA-induced activation of PBMC from healthy donors were also evaluated, and the results are summarized in Table 2. Statistical analysis of the results was performed in comparison to the control PHA stimulation values.

The data demonstrate that wild-type rHBsAg did not have a statistically significant effect on PBMC activation induced by PHA. In contrast, rHBsAg with the G145R mutation significantly suppressed PHA-induced activation of the CD69 molecule on B cells, NK cells, and CD8^+^ T cells. Thus, the immunosuppressive effect of the G145R mutant is stronger than that of the wild-type HBsAg.

### 3.3. Cytokine Secretion by PBMC from Healthy Donors after Stimulation with rHBsAg In Vitro

In addition to evaluation of the expression of activation molecules on immune cells, PBMC activation caused by antigenic stimulation was also assessed by measuring secretion of cytokines IFN-γ, IFN-α, TNF-α, IL-2, and IL-10 to cultured cell supernatants. A summary of the number of donors responding to antigenic stimulation by HBsAg is presented in Table 3. Significant differences were shown only for IL-2 production. Thus, 4 out of 20 donors responded to wild-type rHBsAg, while 18 out of 20 donors responded to the rHBsAg G145R mutant. Thus, PBMC of healthy donors responded to stimulation with the rHBsAg G145R mutant with the production of IL-2 approximately 4.5 times more often compared with wild-type rHBsAg.

**Figure 4:**
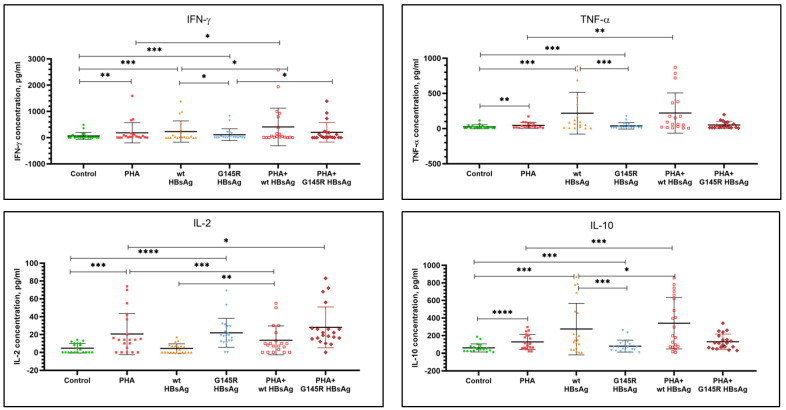
Assessment of cytokine production by PBMC following stimulation with wild-type rHBsAg, the rHBsAg G145R mutant, or PHA, or their combinations. Significant *p* values are reported as asterisks, where * *p* ≤ 0.05, ** *p* ≤ 0.01, *** *p* ≤ 0.001, and **** *p* ≤ 0.0001.

Table 4 and Figure 4 summarize the levels of cytokine response of PBMC from healthy donors to wild-type rHBsAg (ayw2) and rHBsAg G145R mutant (ayw2) stimulation. Statistical analysis of the results was performed in comparison to nonstimulated cells (negative control), cells stimulated with PHA alone, and cells stimulated with rHBsAg alone. The control incubation with PHA caused a statistically significant stimulation of the production of IFN-γ, TNF-α, IL-2, and IL-10 but not IFN-α.

Data in Table 4 indicate that both wild-type rHBsAg and the rHBsAg G145R mutant caused a statistically significant stimulation of the production of IFN-γ, TNF-α, and IL-10, but no effect on IFN-α production. However, the effect of the HBsAg mutant was significantly weaker compared with wild-type HBsAg (*p =* 0.0492 for IFN-γ, *p =* 0.0005 for TNF-α, and *p =* 0.0003 for IL-10). Following stimulation of PBMC with the HBsAg mutant, the levels of cytokines were 2.1 times lower for IFN-γ, 5.7 times lower for TNF-α, and 3.4 times lower for IL-10 compared with stimulation with wild-type HBsAg. In contrast to wild-type rHBsAg, rHBsAg with the G145R mutation caused a statistically significant production of IL-2, and while the amount of secreted IL-2 was low with the average concentration of 22 pg/mL, it was comparable to the level of IL-2 response to PHA stimulation (21 pg/mL). Overall, these findings are consistent with the data on the number of donors responding with IL-2 production to HBsAg stimulation presented in Table 3.

When assessing the effect of rHBsAg on PHA-induced cytokine production by immune blood cells from healthy donors, we took into account that PHA and rHBsAg themselves induce cytokine production to varying degrees. Therefore, the statistical analysis was performed not only for the change in cytokine production in comparison to PHA but also to the recombinant antigens themselves (see Table 4).

Under conditions of PBMC costimulation with PHA, wild-type rHBsAg significantly increased IFN-γ production (2.2 times relative to PHA, *p =* 0.0222; 1.8 times relative to wild-type HBsAg, *p =* 0.0154). Under the same conditions, the rHBsAg G145R mutant added along with PHA had a small trend to increase the IFN-γ level relative to PHA (1.1 times, *p =* 0.2466), but this IFN-γ level was significantly higher compared with stimulation with the rHBsAg G145R mutant alone (1.8 times, *p =* 0.0337). Furthermore, wild-type and mutant rHBsAg had opposite effects on IL-2 production. While wild-type rHBsAg decreased IL-2 relative to PHA 1.5 times (*p =* 0.0002), the mutant rHBsAg enhanced IL-2 level relative to PHA 1.3 times (*p =* 0.0153). On the other hand, costimulation with wild-type or mutant rHBsAg along with PHA increased the IL-2 level 3.15 times for wild-type rHBsAg (*p =* 0.0035) and 1.28 times for the G145R mutant HBsAg (*p =* 0.4004), compared to stimulation with these antigens alone. Wild-type and mutant rHBsAg also differed in their effects on IL-10 production. When assessing the effect of wild-type rHBsAg on PHA-induced cytokine production, a significant increase in the IL-10 level was observed relative to both PHA alone and wild-type rHBsAg alone (2.7 times and 1.2 times, respectively). In contrast, the HBsAg mutant had no effect on PHA-induced IL-10 production by PBMC relative to PHA alone, but increased the IL-10 level 1.6 times (*p* < 0.0001) at assessment relative to the HBsAg mutant alone. For TNF-α, the only significant difference was observed between the combination of wild-type HBsAg with PHA and PHA alone. However, the increased value of TNF-α in this case is almost equal to the value at stimulation of PBMC by wild-type HBsAg alone.

Data on vaccination of donors against hepatitis B were not available. However, preliminary vaccination could form memory cells. This could affect the results of PBMC stimulation. Therefore, a potential correlation was examined between the level of preexisting antibodies to HBsAg in the donor serum (anti-HBsAg status of donor) and the level of cytokines secreted by PBMC after stimulation with wild-type rHBsAg. Table 5 summarizes the numbers of donors that produced cytokines in response to an antigenic stimulation in the groups with different levels of serum anti-HBsAg antibodies, as well as the concentrations of cytokines in culture supernatants in these groups of donors. Results show that there was no significant difference in the cytokine production between donors with anti-HBsAg antibodies at >10 mIU/mL and <10 mIU/mL (Table 5). Thus, the production of cytokines IFN-γ, TNF-α, and IL-10 in response to stimulation with wild-type rHBsAg did not depend on the level of anti-HBsAg antibodies.

Taken together, the results indicate a major difference in immunogenicity between wild-type HBsAg and HBsAg with the G145R mutation, both in the spectrum and level of cytokine production and in the expression profile of activation markers on the surface of immunocompetent cells.

## 4. Discussion

The presented work examined the effect of HBsAg on the activation state of immune cells in vitro. Since a small number of T-cell epitopes in S-HBsAg could be insufficient to induce phenotypic changes in immune cells and cytokine expression, the PBMC cultivation model was used as described in a patent [44]. In the invention, conventional PBMC, before treatment with an immunomodulatory pharmaceutical agent, are precultured for 24–45 h at a high seeding density (up to 10^7^ cells per well in a 24-well plate). After such preincubation, the cultured cells exhibit increased sensitivity, e.g., yield a more powerful and diverse production of cytokines following incubation with the immunomodulatory drug. Without preculturing, especially at a low seeding cell density, PBMC are not capable of such reactions. This effect is facilitated by the intercellular contacts of PBMC, formed during the preliminary cultivation of the cells. For example, it has been shown that the level of expression of surface activation markers CD25 and CD69 is significantly higher in PBMC that have undergone preliminary cultivation with a high seeding density. This approach allows us to partially simulate the in vivo environment in vitro [44].

The current study demonstrated for the first time, using the preculturing model, a major difference in phenotypic and cytokine responses to HBsAg with the G145R mutation compared with wild-type HBsAg in PBMC from healthy donors. The data have shown that wild-type HBsAg, but not the mutant HBsAg, induced an increased expression of the costimulatory molecule CD86 on B cells. The findings for wild-type HBsAg are not consistent with the data by Lobaina et al., who observed an increased expression of CD86 on PBMC following stimulation with a combination of wild-type HBsAg and HBcAg antigens at concentrations of 5 μg/mL each, but not with HBsAg alone at the same dose [45]. The difference in these results can be explained by the experimental conditions between the studies, since we cultured PBMC at a high seeding density for 45 h before antigenic stimulation, in contrast to Lobaina et al. [45], and used a higher HBsAg concentration (20 μg/mL). On the other hand, our results are partly supported by data from another report [46], where dendritic cells (MoDC) of chronically infected HBV patients pulsed with HBsAg at a concentration of 20 μg/mL expressed significantly higher levels of CD86 and CD80 compared with unpulsed MoDC. In this case, the antigen concentration of 20 μg/mL was found to be optimal [46]. Thus, our results indicate that wild-type HBsAg is capable of enhancing the expression of the costimulatory molecule CD86 on antigen-presenting cells, including B cells. This partly explains the development of immune response in people with chronic hepatitis B, in whom the persistence of the virus causes some degree of immunosuppression caused by HBV proteins. For example, HBsAg was shown to be able to be taken up by dendritic cells, and its direct interaction with some components of intracellular signaling complexes leads to blocking the signaling. This results in the formation of immature and tolerogenic populations of dendritic cells with reduced levels of expression of costimulatory molecules, subsequently leading to a weak T-cell immune response [47]. Under these conditions, the upregulation of CD86 expression on B cells following wild-type HBsAg stimulation can serve as a compensatory mechanism facilitating the initiation and development of the T cell immune response. Although the antigen-presenting ability of B cells is much weaker compared with dendritic cells and macrophages, B cells as antigen presenters may also play a special role in the immune response [48,49,50]. Additionally, B cells can successfully phagocyte HBsAg, which can also contribute to the presentation of HBsAg epitopes to T cells and the initiation of a T-cell immune response [51].

The findings from the current study are further supported by observations of the therapeutic effect of prophylactic vaccines. For example, Couillin et al. showed that administration of a standard HBV vaccine as a specific hepatitis B therapy effectively reduced HBV replication and abolished immunological tolerance to HBsAg in approximately 50% of individuals with chronic active HBV replication [52]. The vaccine also induced HBsAg-specific proliferation of PBMC in 25.9% of vaccinated individuals. The immune responses involved CD4^+^ T-lymphocytes, with at least three different epitopes identified. HBV-specific CD4^+^ T-lymphocytes produced high levels of IFN-γ or IFN-α and belonged to the Th1 subpopulation. A decrease in serum HBV DNA in some of the immunized individuals suggests that these CD4^+^ T-cell responses may play a role in controlling viremia during vaccine therapy of chronic HBV carriers [52]. The prophylactic “Combiotech” vaccine, HBsAg of serotype ayw, which was subsequently included in the new multivalent vaccine Bubo^®^-Unigep, also increased the ultimate effectiveness of vaccination when performed in combination with basic management of HBV patients [53,54]. In particular, after a third dose of the vaccine, the percentage of chronic hepatitis B patients with high titers of HBsAg (6000 IU/L and above) decreased 1.4 times (35 ± 11%), and the titer of HBsAg itself decreased accordingly (65 ± 11%). In the group of chronic HBV carriers, the highest HBsAg titer was observed in 34 ± 12% of patients before treatment with the vaccine, and in only one carrier after 12 months after the last vaccine injection. Moreover, at 2 months after the completion of the vaccine therapy, one HBV carrier developed specific serum antibodies at a concentration of 11.2 IU/L [55]. Additionally, immunotherapy of patients with chronic hepatitis B with the “Combiotech” vaccine in combination with basic management positively affected the dynamics of immunological parameters, especially after the third vaccination, which was demonstrated as a decrease in the excessive activation of T and B cells, as well as allergization of the organism and normalization of several metabolic parameters. The immunocorrection included moderate accumulation of T-helper cells, a decrease in the level of B-lymphocytes, and a minimal drop in T-suppressor cells. There was also a significant decrease in the concentration of hepatic transaminase ALT, as well as a trend towards a decrease in bound bilirubin [54]. In the group of patients with chronic hepatitis B who received the vaccine over a 5 month period, the ALT level decreased by 3.9 times, compared with 1.2 times in the control group [55]. In HBV carriers, triple administration of the “Combiotech” vaccine in patients with chronic hepatitis B had a weaker correction effect, with a different spectrum of altered laboratory parameters. The vaccine was shown to increase the number of lymphocytes, stimulate the production of IgA, and decrease the level of T-helper cells [54].

Given the outlined data, our hypothesis that B cells may act as effective antigen-presenting cells during HBV infection may provide a theoretical justification for the observed therapeutic effects of the vaccine in patients with chronic hepatitis B. As such, the activation of B cells with HBsAg can be used for the further development of immunotherapeutic strategies for chronic hepatitis B treatment. At the same time, the inability of HBsAg with the G145R mutation to enhance the expression of CD86 on B cells may indicate a lack of a compensatory mechanism in this escape mutant. In addition, the mutant may exhibit a more potent immunosuppressive effect on the human organism compared with the wild-type virus. The latter, however, requires additional investigation.

The expression of markers CD40 and CD279 was consistent with data reported by Lobaina et al. The authors showed that the wild-type rHBsAg used as a vaccine antigen at a concentration of 5 μg/mL did not increase the expression CD40 on B cells or CD279 on T cells from healthy donors [45]. Here, rHBsAg was used at a concentration of 20 μg/mL and still did not stimulate the expression of these activation markers on T and B cells. The lack of a direct effect of wild-type HBsAg on CD40 and CD279, observed under different experimental conditions, as well as a similar inertness of the escape G145R mutant, seem to demonstrate general properties characteristic of HBV. Interestingly, in contrast to the report by Lobaina et al. [45], we have observed the ability of wild-type HBsAg to induce expression of CD69 on B, T, and NK cells. This suggests increased sensitivity of the cell model selected for the present study. The ability to stimulate the expression of CD69 was also observed for the rHBsAgG145R mutant, although to a lesser degree.

However, under conditions of additional stimulation of PBMC with PHA, HBsAg with the G145R mutation, in contrast to wild-type HBsAg, suppressed PHA-induced expression of CD69 on B cells, NK cells, and CD8^+^ T cells, i.e., the activated state of these cells. This suggests that HBsAg with the G145R mutation can inhibit signaling pathways associated with CD69 expression, which promotes immunosuppression and is utilized by the HBV G145R escape mutant for persistence and spread. We used very mild conditions for PHA stimulation, which are acceptable under modern protocols [56,57]. The conditions we used to stimulate the cells allowed for developing a response both in the direction of amplification and suppression. Therefore, the negative effects associated with cellular memory, when the stimuli that activate resting T lymphocytes initiate apoptotic death in activated T lymphocytes [58,59], were minimized to be as low as possible. The model we have chosen allowed us to fulfill the main goal of the study: assess a difference between two types of HBsAg in their effect on immune cells.

Evaluation of cytokine production by PBMC from healthy donors in response to stimulation with wild-type or G145R mutant rHBsAg also revealed differences between the two antigens. In response to stimulation with wild-type rHBsAg or the rHBsAg G145R mutant, PBMC produced IFN-γ, TNF-α, and IL-10; however, the effect of the rHBsAg mutant was significantly weaker compared to wild-type HBsAg. Nevertheless, production of such a cytokine set is consistent with results of Schlaak et al. seen in PBMC from chronically infected HBV patients [60]. The simultaneous production of the anti-inflammatory cytokine IL-10 and proinflammatory cytokines IFN-γ and TNF-α is not controversial. On the one hand, IL-10 is an immunosuppressive and anti-inflammatory cytokine that inhibits the production of proinflammatory cytokines such as IL-6, IL-12, and TNF-α [61,62]. On the other hand, it has been shown that TNF-α and IL-10 are linked by an autoregulatory loop, in which TNF-α is an inducer of IL-10, and IL-10, in turn, is an inhibitor of TNF-α [63,64,65]. One can assume that the increased production of IFN-γ, TNF-α, and IL-10 in our study may be the result of the activation of memory immune cells, since PBMC donors could have been previously vaccinated against HBV. As is well known, when restimulated with antigens, memory T cells proliferate and differentiate into effector cells much faster than naive T cells [66,67]. For example, antigen-stimulated naive CD4^+^ T cells produce IL-2 as their primary lymphokine, and after priming, develop into effector cells that produce either IFN-γ or TNF-β, as well as IL-2 or IL-4 and related cytokines [68,69,70,71]. In contrast, CD4^+^ memory T cells produce a wide range of cytokines in response to stimulation, including IL-1, IL-2, IL-5, IL-6, IFN-γ, TNF-α, and TNF-β [69,70,71,72,73].

Additionally, the cytokine response to wild-type HBsAg in our study did not depend on the level of antibodies to HBsAg in donor sera, which is consistent with other reports. Previous studies were conducted to determine the correlation of markers of cellular immunity (chemokines and cytokines) with the level of antibody response after vaccination against HBV. Some reports did not reveal such a relationship or the presence of specific markers [74,75,76]. Other authors have shown that PBMC from healthy people vaccinated against HBV responded to stimulation with wild-type rHBsAg with a statistically significant increase in the production of TNF-α, IL-10, and IL-6 32 years after vaccination with plasma vaccine [77] and IFN-γ and IL-2 5 years after vaccination with recombinant vaccines [78], regardless of the levels of anti-HBsAg. For a more thorough assessment of the parameters of cellular immunity against wild-type HBsAg, investigation of PBMC from donors who have never been vaccinated and never had contact with wild-type HBV would be required.

In contrast to wild-type rHBsAg, stimulation of PBMC with rHBsAg with the G145R mutation induced significant production of IL-2. This may indicate the primary HBsAg stimulation of naive T cells. CD4^+^ T cells can skew the immune response towards the Th1, Th2, or Th17 type through the production of various cytokines. Naive CD4^+^ T cells exhibit a limited cytokine response to an antigenic stimulus, and only produce IL-2 before differentiating into various effector cells [68,79,80]. Similarly, the authors in [81] have shown that murine naive CD4^+^ T cells produced only IL-2 on the first day after stimulation with staphylococcal enterotoxin B antigen, but generated IFN-γ and IL-10 following restimulation on days 2 to 7. The development of a primary immune response to the G145R HBsAg mutant, which escapes recognition by neutralizing antibodies, may partly explain the epidemiological situation regarding the mutant spread. Given the reduced immunogenicity of the G145R HBsAg mutant, the mutant spread is likely contained primarily by the cellular immune response.

Secretion of cytokines by T cells in vitro may vary by approaches chosen for T-cell activation. Two of the most widely applied agents for activation of T cells are phorbol 12-myristate 13-acetate (PMA) together with ionomycin or anti-CD3/anti-CD28 stimulation [82]. However, comparative analysis of intracellular cytokines in PBMC stimulated with PMA/ionomycin or PHA demonstrated that the use of PHA for cell activation may limit in vitro artifacts and allow for a more precise analysis of intracellular cytokine production in various disease states [83]. It is generally recognized that PHA primarily activates T cells. However, it is worth considering that PHA has been shown to bind glycoprotein motifs found on the TCR and CD2 of T cells, and to ligate TLRs-2/6, -4, and -5 on monocytes, macrophages, and dendritic cells, antigen-presenting cells essential for T cells to respond to this mitogen [57]. It was interesting to note a partial suppression of PHA-induced production of IL-2 by wild-type HBsAg. PHA binding to the CD2 receptor on the surface of T cells activates various signaling kinases, including JNK. It is the activation of JNK that subsequently triggers the synthesis of IL-2 [84]. Additionally, partial interaction of PHA with the TLR2/6 heterodimer [85] induces the activation of tyrosine and mitogen-dependent kinases, leading to the synthesis of several cytokines, including IL-2 [86,87]. Although activated T cells carrying CD2 are the main source of IL-2, dendritic cells, both plasmacytoid and myeloid, that express TLR receptors including TLR2/6, can also produce IL-2 [88,89]. Similar results were obtained for murine dendritic cells of various subtypes, including Langerhans cells [90,91]. Recent studies have shown that HBsAg can block TLR2 signaling by inhibiting phosphorylation of JNK-1/2 and c-Jun kinases [92,93]. Apparently, this mechanism is also involved in the inhibition of PHA-induced production of IL-2 by wild-type HBsAg. In contrast, the mutant G145R HBsAg, which enhanced PHA-induced IL-2 production, does not appear to be able to inhibit the phosphorylation of JNK-1/2 and c-Jun kinases.

The increase in IFN-γ production in response to costimulation with PHA and wild-type HBsAg can be explained by the cumulative effect, since both PHA and wild-type HBsAg individually also induced IFN-γ production by PBMC from healthy donors. In contrast, there was a small cumulative effect in the presence of the G145R rHBsAg mutant. Both rHBsAg antigens also differed in their effects on PHA-induced IL-10 production. This confirms the major differences between the two antigens in the immunological reactions they trigger.

Overall, the major differences between the immunopotentiation effects of wild-type and G145R mutant HBsAg emphasize the importance of including mutant HBsAg in the composition of a multivalent vaccine. However, these differences question the compatibility of wild-type and mutant HBsAg antigens as components of a complex vaccine. In the presented work, we studied the effects of these antigens on immune cells separately. The reactions of PBMC to costimulation with these antigens in vitro are a subject of further research. However, available data suggest the antigens’ compatibility, which has promising prospects. Comparative studies of sera from people who were vaccinated with monovalent vaccines and those recovered from HBV infection have demonstrated that only sera of the recovered patients contained highly active antibodies against both the G145R mutant HBsAg and wild-type HBsAg. Yet, the patients’ sera varied considerably in the level of specific antibodies to the G145R mutant HBsAg [94]. Since the G145R mutant can persist as minor subpopulations and can coexist with wild-type HBV, it is likely that patients that recovered from HBV may develop antibodies to both viruses simultaneously. As such, a vaccine containing both wild-type and the G145R mutant HBsAg can also confer immunity to both types of the virus. This is supported by our earlier studies of antigens of the Bubo^®^-Unigep vaccine in vivo in mice and sheep, where the HBsAg variants were tested both separately and together [39]. The experiments have shown that simultaneous immunization of animals with three recombinant HBsAg variants, including, in addition to the G145R mutant, wild-type ad and ay HBsAg immobilized on aluminum hydroxide, elicited the production of antibodies that recognized natural wild-type and G145R mutant-type viral particles. The level of antibodies to the G145R mutant antigen in sheep was higher than in the mouse model [39]. Studying the immune response to the combined vaccine antigen in humans remains to be of great importance. This is expected to be partially assessed in ongoing Phase III clinical trials of the Bubo^®^-Unigep vaccine, but requires additional in-depth studies, including post-registration evaluation.

## 5. Conclusions

The results of the current study have demonstrated that wild-type rHBsAg induces expression of the costimulatory molecule CD86 and the activation marker CD69 on B cells and expression of CD69 on T cells (CD4^+^ and CD8^+^) and NK cells. Furthermore, wild-type rHBsAg induces production of TNF-α, IL-10, and IFN-γ, but not IL-2, in PBMC from healthy donors in vitro, which is independent of the anti-HBsAg status of donors. Under coculture conditions, wild-type rHBsAg does not affect the expression levels of surface cell markers, although it enhances secretion of IFN-γ and IL-10 and simultaneously inhibits production of IL-2 by immune cells induced by PHA. The rHBsAg G145R mutant induces expression of CD69 on B, NK, and T cells (both CD4^+^ and CD8^+^), similarly to wild-type rHBsAg, although the effect is weaker. However, in contrast to wild-type rHBsAg, rHBsAg with the G145R mutation does not affect the expression of CD86 on B cells, and induces IL-2 along with lower production of TNF-α, IL-10, and IFN-γ. Furthermore, unlike wild-type rHBsAg, the mutant also suppresses PHA-induced activation of the CD69 molecule on B cells, NK cells, and CD8^+^ T cells, has a weaker effect on IFN-γ and IL-10 production, and displays an opposite effect on IL-2 production under coculture conditions. Neither wild-type rHBsAg nor the rHBsAgG145R mutant affects CD40 and HLA-DR expression on B cells or HLA-DR and CD279 expression on T cells and NK cells.

The distinct immunogenic properties of the G145R mutant demonstrated in vitro in this study, together with its previously described antigenic and morphological characteristics and the recognized profile of the specific antibody production in vivo, provide strong support toward including the mutant antigen in the composition of HBV vaccines and further exploring the mechanisms of the vaccinal G145R escape mutant interaction with various components of the immune system. In addition, the development of antibodies after vaccination of humans with a multivalent vaccine containing HBsAg with the G145R mutation needs to be evaluated. Similar assessments can also be performed for other HBV variants. The results will improve the understanding of both the pathogenesis and virology of clinically significant HBV mutants, as well as contribute to HBV vaccine prophylaxis and the development of therapeutic vaccines.

## Figures and Tables

**Table 1:** Characteristics of PBMC isolated from the buffy coats of healthy donors.

Cell Population	Relative Amount, %	Reference Values for Blood, %
T cells	71.56	61–85
B cells	9.39	7–17
CD8^+^ T cells (CTL)	30.44	19–35
CD4^+^ T cells (Th)	65.29	35–65
NK cells	8.56	8–18
NKT cells	4.03	0.5–6
Neutrophils	1.73	37–63
Eosinophils	0.44	0–3
Monocytes	8.59	2–9
Immunoregulatory index CD4^+^/CD8^+^	2.30	1.5–2.6

Data shown represent mean values for 20 donors. CTL, cytotoxic T lymphocytes; Th, T-helper cells.

**Table 2:** Effects of stimulation of PBMC from healthy donors with wild-type rHBsAg (ayw2) and the rHBsAg G145R mutant (ayw2) ^1^.

Activation Markers	Mean Value ± SEM						*p*-Value, Group vs. Negative Control			*p*-Value, Group vs. PHA	
	Negative Control	PHA	wt HBsAg	G145R HBsAg	PHA+ wt HBsAg	PHA+ G145R HBsAg	PHA	wt HBsAg	G145R HBsAg	PHA+ wt HBsAg	PHA+ G145R HBsAg
B-cell activation; % cells for CD69 and CD86, MFI for CD40 and HLA-DR											
CD69	6.11 ± 1.34	18.84 ± 3.98	8.83 ± 2.23	7.24 ± 1.46	18.91 ± 4.17	13.62 ± 2.65	<0.0001 (*)	0.0073 (*)	0.0086 (*)	0.5459 (ns)	0.0010 (*)
CD86	3.08 ± 0.17	3.76 ± 0.31	4.13 ± 0.32	3.38 ± 0.25	4.07 ± 0.47	4.11 ± 0.42	0.0133(*)	0.0001 (*)	0.0570 (ns)	0.2413 (ns)	0.4749 (ns)
CD40	23.64 ± 0.84	26.51 ± 1.50	23.88 ± 0.86	23.34 ± 0.82	26.25 ± 1.50	26.26 ± 1.36	0.0139 (*)	0.5706 (ns)	0.1117 (ns)	0.3488 (ns)	0.2815 (ns)
HLA-DR	48.05 ± 6.77	81.20 ± 14.79	56.01 ± 8.72	52.08 ± 6.56	84.00 ± 15.26	74.66 ± 14.37	<0.0001 (*)	0.1084 (ns)	0.0898 (ns)	0.6397 (ns)	0.4951 (ns)
NK-cell activation; % cells											
CD69	25.11 ± 4.37	39.61 ± 5.08	31.21 ± 6.03	26.86 ± 4.75	39.23 ± 5.64	34.11 ± 4.47	<0.0001 (*)	0.0010 (*)	0.0385 (*)	0.9323 (ns)	<0.0001 (*)
T-cell activation; % cells											
HLA-DR	5.90 ± 0.99	7.87 ± 1.12	5.82 ± 0.99	6.30 ± 1.27	—	—	0.0045 (*)	0.5509 (ns)	0.7578 (ns)	—	—
CD4+ T-cell activation; % cells											
CD69	1.70 ± 0.44	21.72 ± 5.00	2.74 ± 0.83	2.08 ± 0.50	23.64 ± 5.14	18.92 ± 4.10	<0.0001 (*)	0.0210 (*)	0.0104 (*)	0.0637 (ns)	0.1231 (ns)
CD279 (PD-1)	3.62 ± 0.46	3.58 ± 0.48	3.72 ± 0.52	3.51 ± 0.46	3.56 ± 0.48	3.33 ± 0.47	0.5039 (ns)	0.4123 (ns)	0.6410 (ns)	0.6215 (ns)	0.0896 (ns)
CD8+ T-cell activation; % cells											
CD69	4.80 ± 1.21	21.94 ± 4.66	7.28 ± 1.65	5.81 ± 1.15	22.29 ± 5.02	17.36 ± 3.95	<0.0001 (*)	0.0012 (*)	0.0391 (*)	0.8408 (ns)	0.0001 (*)
CD279 (PD-1)	4.07 ± 0.69	3.77 ± 0.68	4.18 ± 0.69	4.32 ± 0.72	3.88 ± 0.66	3.55 ± 0.67	0.8052 (ns)	0.5706 (ns)	0.5217 (ns)	0.9273 (ns)	0.2492 (ns)

^1^ wt, wild type; PHA, phytohemagglutinin; vs, versus; * significant difference; ns, nonsignificant difference; MFI, mean fluorescent intensity.

**Table 3:** Numbers of donors responding to PBMC stimulation with wild-type rHBsAg (ayw2) and the rHBsAg G145R mutant (ayw2) and PBMC cytokine production ^1^.

Cytokine	N of Tested Donors	Antigen for PBMC Stimulation						*p*-Value (Responders), wt HBsAg vs. G145R HBsAg
		wt HBsAg			G145R HBsAg			
		N of Responders	% of Total Donors	Cytokine Range, pg/mL	N of Responders	% of Total Donors	Cytokine Range, pg/mL	
IFN-γ	20	13	65	0–1390	14	70	0–820	>0.9999 (ns)
IFN-α	20	1	5	0–2	0	0	0–0	>0.9999 (ns)
TNF-α	18	15	83	5–890	16	89	5–174	>0.9999 (ns)
IL-2	20	4	20	0–17	18	90	0–69	<0.0001 (*)
IL-10	20	15	75	8–870	17	85	7–260	0.6948 (ns)

^1^ vs., versus; wt, wild type; * significant difference; ns, nonsignificant difference.

**Table 4:** Cytokine production in response to stimulation of PBMC from healthy donors with wild-type rHBsAg (ayw2) and the rHBsAg G145R mutant (ayw2) ^1^.

Cytokine	Mean Value of Cytokine Concentration (pg/mL) ± SEM						*p*-Value						
							Group vs. Negative Control			Group Vs. PHA		PHA+ wt HBsAg vs. wt HBsAg	PHA+ G145R HBsAg vs. G145R HBsAg
	Negative Control	PHA	wt HBsAg	G145R HBsAg	PHA+ wt HBsAg	PHA+ G145R HBsAg	PHA	wt HBsAg	G145R HBsAg	PHA+ wt HBsAg	PHA+ G145R HBsAg		
IFN-γ	65.75 ± 29.63	186.60 ± 86.37	232.05 ± 91.08	111.95 ± 50.28	408.60 ± 160.43	204.10 ± 84.03	0.0020 (*)	0.0005 (*)	0.0001 (*)	0.0222 (*)	0.2466 (ns)	0.0154 (*)	0.0337 (*)
IFN-α	0.00 ± 0.00	0.00 ± 0.00	0.10 ± 0.10	0.00 ± 0.00	035 ± 0.35	0.00 ± 0.00	—	>0.9999 (ns)	—	>0.9999 (ns)	—	>0.9999 (ns)	—
TNF-α	24.78 ± 6.66	45.22 ± 10.04	218.39 ± 69.95	38.50 ± 10.83	220.83 ± 67.57	50.44 ± 12.18	0.0038 (*)	0.0002 (*)	0.0009 (*)	0.0038 (*)	0.2477 (ns)	0.9278 (ns)	0.1710 (ns)
IL-2	4.65 ± 1.19	20.50 ± 5.13	4.30 ± 1.17	21.85 ± 3.63	13.55 ± 3.60	28.00 ± 5.11	0.0002 (*)	0.6289 (ns)	<0.0001 (*)	0.0002 (*)	0.0153 (*)	0.0035 (*)	0.4004 (ns)
IL-10	59.25 ± 10.43	127.10 ± 19.12	274.15 ± 65.12	79.50 ± 15.20	340.40 ± 65.52	129.80 ± 19.35	<0.0001 (*)	0.0002 (*)	0.0004 (*)	0.0007 (*)	0.8517 (ns)	0.0137 (*)	<0.0001 (*)

^1^ PHA, phytohemagglutinin; vs., versus; wt, wild type; * significant difference; ns, nonsignificant difference; —, not determined.

**Table 5:** Comparison of PBMC cytokine responses to stimulation with wild-type rHBsAg (ayw2) in donor groups with different levels of serum antibodies to HBsAg ^1^.

Cytokine	Group 1 (*n* = 7), Anti-HBsAg < 10 mIU/mL				Group 2 (*n* = 9), Anti-HBsAg > 10 mIU/mL				*p*-Value	
	Responders		Cytokine Concentration, pg/mL		Responders		Cytokine Concentration, pg/mL		Responders	Cytokine Concentration
	Number	% of Total	M ± m	Range	Number	% of Total	M ± m	Range		
IFN-γ	5	71	212.86 ± 136.10	0–990	6	67	324.22 ± 172.74	0–1390	>0.9999 (ns)	0.7535 (ns)
IFN-α	0	0	0.00 ± 0.00	0–0	1	11	0.22 ± 0.22	0–2	>0.9999 (ns)	—
TNF-α	5	71	194.86 ± 94.94	6–689	8	89	274.56 ± 119.33	5–890	0.5500 (ns)	0.7577 (ns)
IL-2	2	29	6.43 ± 2.17	0–17	2	22	4.56 ± 1.75	0–13	>0.9999 (ns)	0.4751 (ns)
IL-10	5	71	319.14 ± 121.71	19–780	8	89	335.00 ± 101.75	41–870	0.5500 (ns)	0.5360 (ns)

^1^ ns, nonsignificant difference; —, not determined.

## Data Availability

A description of the Bubo^®^-Unigep vaccine clinical trials is available at: https://grls.rosminzdrav.ru/CiPermitionReg.aspx?PermYear=0&DateBeg&DateEnd&DateInc&NumInc&RegNm&Statement&Protocol&Qualifier&ProtoNum&idCIStatementCh&CiPhase&RangeOfApp&Torg=%D0%B1%D1%83%D0%B1%D0%BE&LFDos&Producer&Recearcher&sponsorCountry&MedBaseCount&CiType&PatientCount&OrgDocOut=2&Status&NotInReg=0&All=0&PageSize=8&order=date_perm&orderType=desc&pagenum=1 (accessed on 18 October 2021).

## Supplementary Materials

The following supporting information can be downloaded at: https://www.mdpi.com/article/10.3390/vaccines10020235/s1, Figure S1: Gating used for immunophenotyping of PBMC from healthy donors; Figure S2: Gating strategy used in assessment of T cell activation; Figure S3: Gating strategy used in assessment of B cell activation; Figure S4: Gating strategy used in assessment of T/B/NK cell activation.
