# Supplementary material for: Recombinant HBsAg of the Wild-Type and the G145R Escape Mutant, included in the New Multivalent Vaccine against Hepatitis B Virus, Dramatically Differ in their Effects on Leukocytes from Healthy Donors In Vitro
Source: Vaccines (Basel). 2022 Feb 3;10(2):235. doi: 10.3390/vaccines10020235 (PMC8880183; doi:10.3390/vaccines10020235)
Supplement: Supplementary file 1 [file vaccines-10-00235-s001.zip › vaccines-1518139-supplementary.pdf]

GATING STRATEGIES

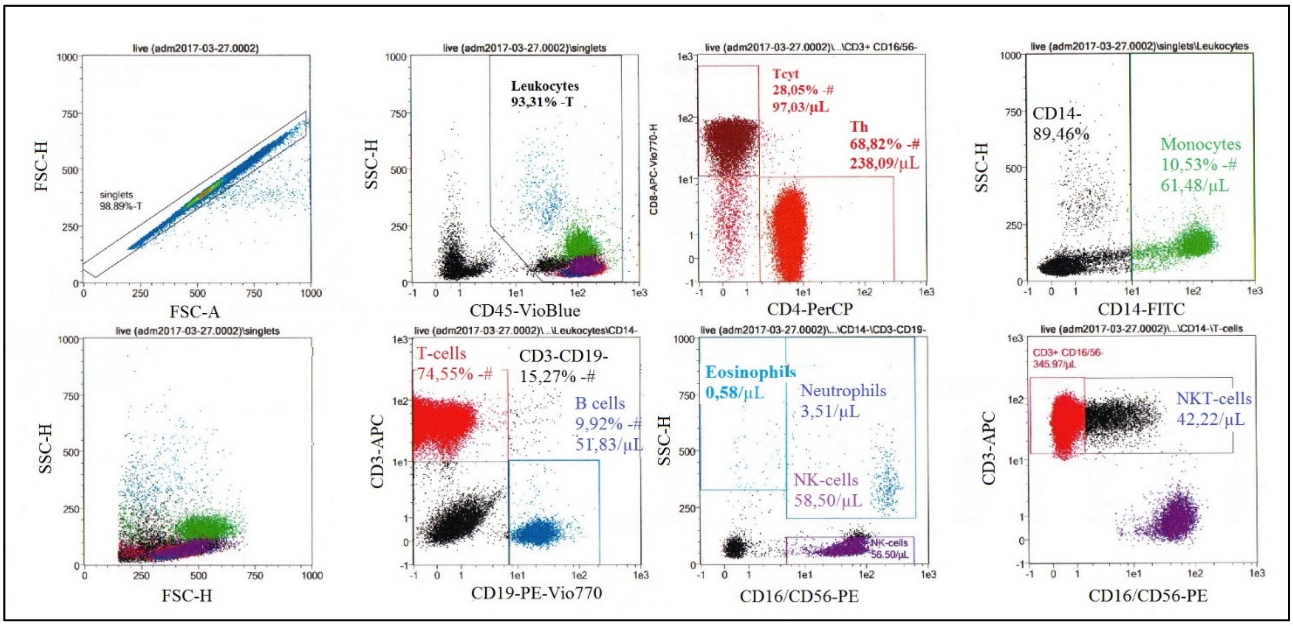

Figure S1. Gating used for immunophenotyping of PBMC from healthy donors.

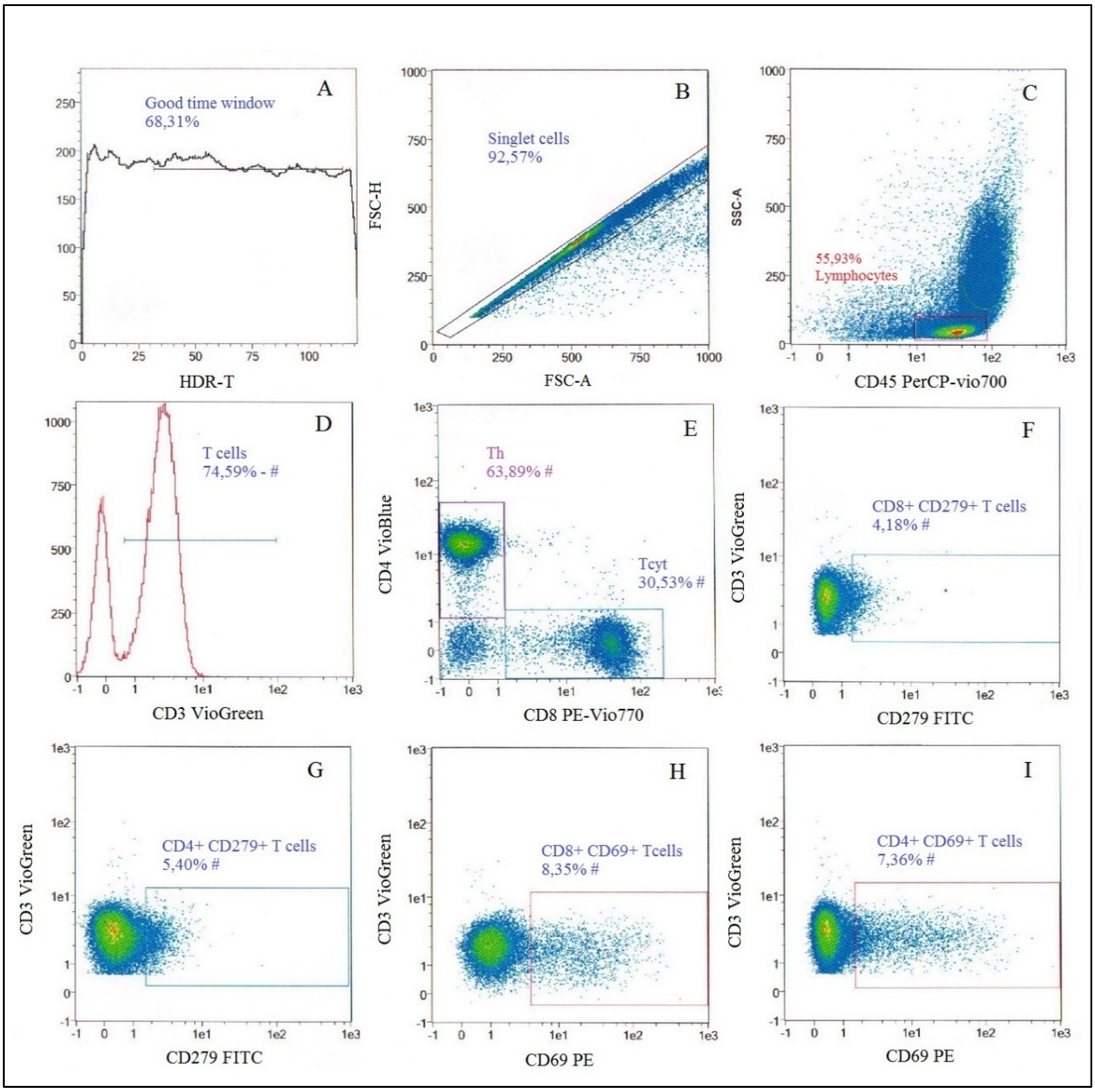

Figure S2. Gating strategy used in assessment of T cell activation. (**A**) Determination of a cytometry time interval with a steady sample feed; (**B**) Exclusion of clumping cells using a FSC-A/FSC-H diagram; (**C**) Selection of lymphocytes by expression of CD45; (**D**) Selection of T cells by expression of CD3, using a histogram of fluorescence intensity; (**E**) Separation of T cells into two subpopulations: CD4+ T-cells (Th) and CD8+ T cells (Tcyt); (**F**) Analysis of CD279 (PD-1) expression on CD8+ T cells; (**G**) Analysis of CD279 (PD-1) expression on CD4+ T cells; (**H**) Analysis of CD69 expression on CD8+ T cells; (**I**) Analysis of CD69 expression on CD4+ T cells.

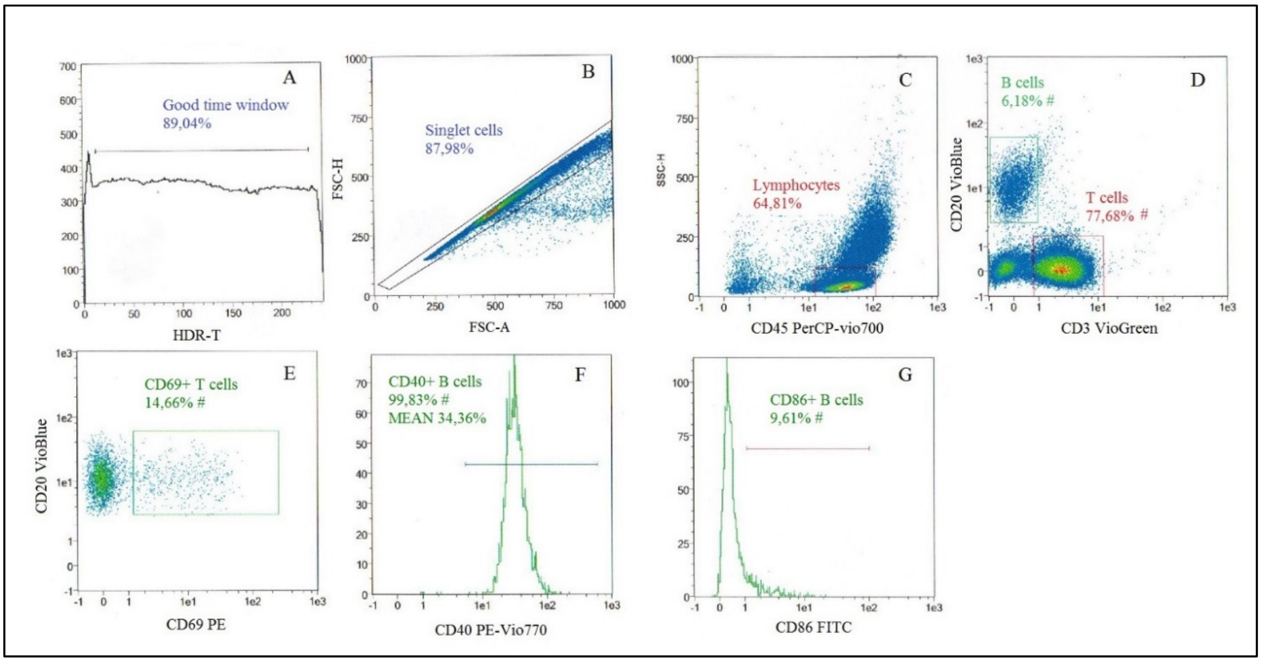

Figure S3. Gating strategy used in assessment of B cell activation. (**A**) Determination of a cytometry time interval with a steady sample feed; (**B**) Exclusion of clumping cells using a FSC-A/FSC-H diagram; (**C**) Selection of lymphocytes by expression of CD45; (**D**) Selection of T cells by expression of CD3 and B cells by expression of CD20; (**E**) Analysis of CD69 expression on B cells; (**F**) Analysis of CD40 expression on B cells, using a histogram of fluorescence intensity; (**G**) Analysis of CD86 expression on B cells, using a histogram of fluorescence intensity.

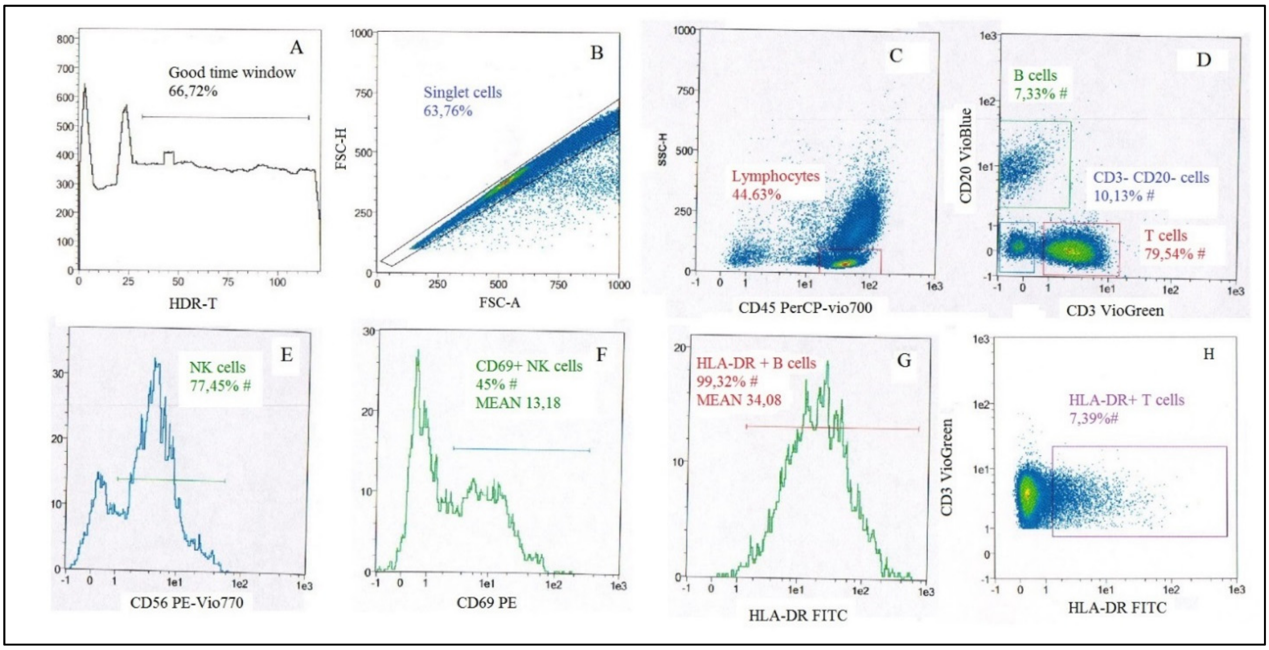

Figure S4. Gating strategy used in assessment of T/B/NK cell activation. (**A**) Determination of a cytometry time interval with a steady sample feed; (**B**) Exclusion of clumping cells using a FSC-A/FSC-H diagram; (**C**) Selection of lymphocytes by expression of CD45; (**D**) Selection of T cells by expression of CD3 and B cells by expression of CD20; (**E**) Selection of NK cells by expression of CD56, from the gate of $CD3^-$ $CD20^-$cells; (**F**) Analysis of CD69 expression on NK cells, using a histogram of fluorescence intensity; (**G**) Analysis of HLA-DR expression on B cells, using a histogram of fluorescence intensity; (**H**) Analysis of HLA-DR expression on T cells.
